# Ventilatory support in neonates with cancer: balancing oxygen and oncologic stability

**DOI:** 10.1097/MS9.0000000000004238

**Published:** 2025-10-30

**Authors:** Asra Amjad, Mir Raza Ali, Iman Osman Abufatima

**Affiliations:** a Department of Medicine, Islamic International Medical College, Riphah International University, Rawalpindi, Pakistan; b Department of Medicine, Jinnah Postgraduate Medical Centre, Karachi, Sindh, Pakistan; c Department of Medicine, University of Medical Sciences and Technology, Khartoum, Sudan

*To the Editor,*

As perinatology, diagnostics, and newborn intensive care progress, it is becoming more common to diagnose congenital malignancies in the neonatal period, including congenital leukemia, neonatal neuroblastoma, and teratomas. Neonatologists are being challenged to maintain life under ever-more complicated circumstances, as seen by the sharp increase in the use of neonatal intensive care services for severely preterm and frail children in recent years^[1]^.

In this changing environment, we face a conundrum when a newborn has both cancer and respiratory failure: the very ventilatory techniques that sustain life may affect tumor biology through inflammatory and redox pathways. Because of their undeveloped lungs, weak antioxidant defenses, and increased susceptibility to oxygen exposure, neonates are particularly sensitive to oxidative damage. At the same time, new research in oncology portrays reactive oxygen species as active contributors to carcinogenesis, DNA damage induction, and the modification of cancer cell survival pathways, rather than merely as harmful bystanders^[2,3]^.

In this way, oxygen may function as a “double-edged drug” in the newborn oncologic context, maintaining critical tissue oxygenation while, if administered in excess, meeting the redox requirements of malignant clones. Furthermore, mechanical ventilation is not innocuous in and of itself. According to experimental and translational research, ventilator-induced lung injury causes local and systemic inflammatory cascades through cyclic stretch and alveolar overdistension. These cascades may foster a tumor-permissive microenvironment, which in turn encourages invasiveness and metastasis in lung models^[4,5]^.

In effect, we run the danger of exposing a newborn whose tumor and host milieu are in delicate balance to two pro-oncogenic exposures: oxidative burden from high FiO_2_ and inflammation caused by mechanical stress. Although current neonatal oncology case reports and series highlight the importance of supportive care and the timing of chemotherapy in the face of organ immaturity, they do not explicitly distinguish ventilatory parameters as independent predictors of oncologic outcomes, making it difficult to translate the mechanistic theory into clinical reality^[6]^.

But in those accounts, episodes of respiratory collapse, prolonged ventilation, and high oxygen demand frequently lead to tough choices regarding the severity of therapy and whether to stay with full life support or move toward comfort-based methods. In light of this, a clinical framework that makes sense is required. In order to reduce the danger of hyperoxia and prevent hypoxic injury, we should first implement conservative oxygenation objectives, which are similar to the standard newborn approach that favors SpO_2_ targets in the 90–95% range^[7]^. Such “precision oxygenation” is not just cautious in cancerous newborns; it may be a tactic to lessen unintentional redox-driven tumor stress. Second, ventilator techniques that prioritize lung protection include modest tidal volumes, careful PEEP titration, limited effective FiO_2_, and, when practical, early weaning. These methods lessen inflammatory signaling brought on by strain, which could support systemic pro-tumor pathways^[5]^.

Third, ventilatory decisions should be made with a multidisciplinary perspective rather than in isolation. When determining ventilatory intensity, neonatologists, pediatric oncologists, respiratory therapists, and palliative care teams must collaborate to consider family values, long-term oncologic prognosis, and short-term survival. This linked logic easily leads to three research goals for the future. In order to facilitate correlative and multivariate modeling of ventilation–tumor interactions, prospective registries should incorporate ventilatory metadata (FiO_2_ exposure, duration, and pressures), serial biomarkers of redox stress and inflammation, and oncologic endpoints.

Targeted antioxidant adjuncts, such as glutathione precursors or clinically appropriate scavengers, may be tested in parallel translational studies to see if they alter the way ventilator-induced oxidative stress affects tumor cell behavior. In order to achieve an “onco-respiratory equilibrium” in newborns, we need to lastly investigate adaptive, AI-driven ventilatory systems that dynamically titrate support depending on real-time metabolic, oxidative, and physiological inputs.

In conclusion, newborns with cancer make us reevaluate our presumptions since in these cases, ventilation and oxygen are active biologic exposures that may affect tumor behavior rather than passive modalities. Clinicians should make ventilatory decisions as part of a cohesive oncologic treatment plan, document ventilatory exposures meticulously, and fall back on lung-protective, conservative oxygenation techniques until solid data become available. In order to establish evidence-based ventilatory guidelines that respect both survival and oncologic stability, the neonatal oncology and critical-care groups must collaborate. We can only hope to maintain life without unintentionally causing the very illnesses we are trying to eradicate if we work together.

This manuscript complies with TITAN Guidelines 2025, declaring no use of AI^[8]^.
